# Maternal and paternal genetic variation in Estonian local horse breeds in the context of geographically adjacent and distant Eurasian breeds

**DOI:** 10.1111/age.12835

**Published:** 2019-09-02

**Authors:** E. Sild, S. Värv, T. Kaart, J. Kantanen, R. Popov, H. Viinalass

**Affiliations:** ^1^ Institute of Veterinary Medicine and Animal Sciences Estonian University of Life Sciences Kreutzwaldi 1 Tartu 51014 Estonia; ^2^ Natural Resources Institute Finland (Luke) Myllytie 1, Alimentum Jokioinen FI‐31600 Finland; ^3^ Yakutian Research Institute of Agriculture (FGBNU Yakutskij NIISH) ul. Bestyzhevo‐Marlinskogo 23/1 Yakutsk 677001 The Sakha Republic (Yakutia), Russia

**Keywords:** Baltic Sea region, Estonian local horse breeds, mtDNA, Siberia, Y chromosome

## Abstract

The maternal and paternal genetic variation of horse breeds from the Baltic Sea region, including three local Estonian breeds, was assessed and compared with that of Altai and Yakutian horses. In the mtDNA D‐loop region, 72 haplotypes assigned to 20 haplogroups in the nine breeds were detected. In Estonian local breeds, 38 mtDNA haplotypes were found, and five of them were shared by the three breeds. More than 60% of all identified haplotypes were rare. Compared with the Estonian Native and Estonian Heavy Draught breeds, a higher haplotypic diversity was found in the Tori breed (*h* = 0.969). Moreover, four haplotypes shared among Finnish and Estonian local horse breeds indicated ancient ancestry, and of these, H30 (haplogroup D3) showed global sharing and genetic links between modern Baltic Sea region and Siberian horses, specifically. The studied breed set showed high variability in maternal inheritance and mixed patterns of the international and native breeds of the Siberian and Baltic regions. No variation was found in paternally inherited markers among horse breeds in the Baltic Sea region.

Genetic characterization of farm animal breeds is an important task in the conservation of animal genetic resources at the national and international levels. Variations in mtDNA and Y chromosomal markers in farm animals have been used to investigate domestication, phylogenetic relationships among populations, and temporal and spatial changes in maternal and paternal lineages (Kantanen *et al*. 2009; Rannamäe *et al*. 2016). Genetic variation in mitogenomes is typically interpreted as reflecting the survival of ancestral variability in modern breeds rather than being a result of animal breeding and artificial selection (Cieslak *et al*. 2010). Although high mtDNA variation in horse breeds has been observed in terms of haplotype and haplogroup diversity, the Y chromosomal variation has been low, and it appears that very few stallions contributed to the paternal genetic diversity of horses (Lindgren *et al*. 2004; Wallner *et al*. 2004, 2017; Brandariz‐Fontes *et al*. 2013; Kreutzmann *et al*. 2014). Comparative analysis of male‐specific diversity based on sequencing data in American, Asian and European breeds detected that the Asian breeds in particular showed more male lineages compared with the European breeds (Felkel *et al*. 2018).

Based on the local native horse population in the Estonian territory in the nineteenth century, three different breeds were developed, and of these, the Estonian Native breed has maintained some indigenous characteristics (e.g. dorsal stripe and smaller body size compared with the Tori and Heavy Draught breeds). As described by Petersen *et al*. (2013) and Sild *et al*. (2019), the genetic relationship between North European and Central Siberian horses exists on the basis of autosomal markers. Here, our aim was to investigate genetic diversity and relationships using maternal and paternal genetic markers in Estonian, Finnish, Latvian and Siberian (Altai and Yakutian) breeds.

Hair or blood samples were collected from 259 horses of nine breeds (the pedigree information for the Altai horse was not known, the samples from Yakutian horses were collected from three different villages and the horses from the rest of the breeds were unrelated considering at least two generations; Table S1) and analysed for the mtDNA D‐loop sequence and five loci of Y chromosome‐specific microsatellites (Table S2). For mtDNA D‐loop sequences, the alignment and nucleotide position determination were based on reference sample X79547 (Xu & Arnason 1994). genaiex 6.5 (Peakall & Smouse 2012) was used to detect haplotypes. The haplogroups were identified using the nomenclature by Cieslak *et al*. (2010). network version 4.6.1.1 (Bandelt *et al*. 1995) was used to construct a phylogeny network of haplogroups. The diversity parameters, including haplotype diversity (Fu & Li 1993) and nucleotide diversity (Jukes & Cantor 1969; Lynch & Crease 1990), were estimated using dnasp version 5.1 (Librado & Rozas 2009). Deviations of empirical nucleotide diversity estimates from expected values were assessed using Tajima's *D*‐test (Kimura 1983). Euler diagrams were constructed to illustrate the overlapping of common and unique haplotypes among breeds.

The sequenced mtDNA (GeneBank accession nos MH794668–MH794767, MH794769–MH794847, MH794849–MH794928) D‐loop region contained 44 SNPs and one deletion at the mtDNA genome position 15528 (Table S3).

The paternal Y chromosomal microsatellites showed no variation in breeds from the Baltic Sea region and the Altai breed, but two alleles were detected in the Yakutian Horse at marker YA16 (Table S4). This male‐specific population split is consistent with the hypothesis by Felkel *et al*. (2018) that the Yakutian Horse carries Y haplotypes from different haplogroups and the breed originates from many sources. The observed paternal genetic uniformity is, in general, in concordance with previous studies (Lindgren *et al*. 2004; Wallner *et al*. 2004; Brandariz‐Fontes *et al*. 2013; Kreutzmann *et al*. 2014). In more recent studies, analyses using Y chromosome‐specific high‐resolution haplotyping have revealed more variation. However, modern European breeds clustered together as one group and exhibited the influence of Oriental stallions (Wallner *et al*. 2017; Felkel *et al*. 2018).

Seventy‐two different mtDNA haplotypes were detected in the nine breeds analysed (Table S5). Furthermore, the number of haplotypes in one breed varied from seven (Altai) to 25 (Tori) (Table 1), reflecting the different numbers of mare lines that have contributed to the gene pools of the present breeds. The prevalent haplotype H30 (29 individuals of total 259) was not found in the Latvian and Trakehner breeds and was rare in the Tori horse. The same haplotype predominated in the Yakutian breed (frequency 0.44).

**Table 1:** mtDNA diversity estimates in the analysed breeds

Breed	*n*	*n*_H_	*n*_unique_	*h*	*π*	Tajima's *D*
Altai	11	7	3	0.909	0.017	0.03
Arabian	29	9	1	0.882	0.016	0.64
Estonian Heavy Draught	30	13	3	0.871	0.011	−0.66
Estonian Native Horse	40	19	6	0.947	0.016	0.43
Finnhorse	37	18	6	0.926	0.016	−0.04
Latvian	22	17	8	0.978	0.018	0.15
Tori	40	25	8	0.969	0.015	−0.08
Trakehner	23	16	5	0.964	0.016	0.24
Yakutian	27	11	6	0.789	0.012	−0.13
All	259	72	–	0.964	0.016	−0.09

*n*, sample size; *n*_H_, number of haplotypes; *n*_unique_, number of unique haplotypes; *h*, haplotype diversity; *π*, nucleotide diversity.

In the entire dataset, the mean haplotype diversity *h* was 0.964 and the mean nucleotide diversity *π* was 0.016 (Table 1). The most variable breed was the Latvian Horse (*h *=* *0.978 and *π *= 0.018). Within the Estonian local population, a smaller maternal genetic variation was revealed in the Estonian Heavy Draught breed, which has a small population size (*n* = 350). The low diversity can be explained by random genetic drift and recent bottlenecks. Although the Tajima's *D*‐test results showed agreement with the neutral mutation model, the lowest (−0.70, Estonian Heavy Draught) and highest (0.43, Estonian Native) values indicated differences in population genetic processes.

Focusing only on Estonian breeds, it follows that, of the 110 horses representing Estonian Native, Estonian Heavy Draught and Tori breeds, 42 (38%) carried one of the five haplotypes shared (Fig. 1a, b). There were 38 different haplotypes in the three Estonian local breeds. In total, 63% of the haplotypes were rare and detected in only one or two horses; of these haplotypes, the highest number was counted in the Tori breed (14; Fig. 1b, Table S5). The variation in modern Estonian local breeds is probably, to some extent, due to the preference for various mare types at the breed's formation stage.

**Figure 1:**
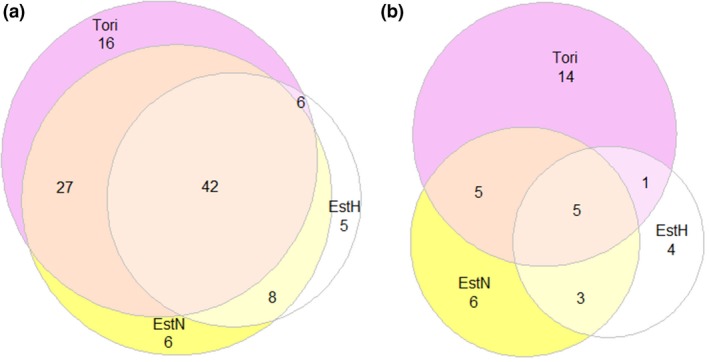
Euler diagrams of Estonian local breeds presenting (a) the number of horses according to their haplotype shared by or unique to different breeds and (b) the number of haplotypes common or unique to different breeds (EstN, Estonian Native Horse; EstH, Estonian Heavy Draught; Tori, Tori Horse).

For both the Estonian Native and Estonian Heavy Draught, the breeds’ gene pools have been influenced by major declines in numbers. In the case of the Heavy Draught, there has also been some introgression from other cold‐blooded breeds, and for the Estonian Native, one of the influencing factors has been temporal population expansion.

In total, 20 haplogroups were determined (Table S6), and the number of haplogroups ranged from seven (Altai and Yakutian) to 14 (Tori and Trakehner). One haplogroup (D3) was common in all nine breeds and was the most frequent in the Yakutian Horse (44%). Haplogroup D3 with F, B1 and X3c1 included five common haplotypes shared among the Estonian local breeds.

The occurrence of haplogroups A, B1 and X3c1 could be interpreted as a genetic contribution of the oldest/ancient native matrilines in the Baltic Sea region. Dating back to the first archaeological equine findings in Estonia, the sequences in these horses determined by Vilà *et al*. (2001) corresponded most likely to haplogroup X3c1. Haplogroup X3c1 (appearing in 1500 BC according to Cieslak *et al*. (2010) showed a similar proportion (12–17%) in Estonian local breeds and the Latvian Horse. The constructed haplogroup network (Fig. S1) showed the grouping of horses from both native and international breeds into the same haplogroups.

None of the studied breeds showed a high frequency of unique alleles and genetic distinctiveness in the mtDNA data, but the distribution of haplotypes revealed relationships among geographically distant breeds. Only one haplotype (H18 of haplogroup F) was shared across all local breeds in the Baltic Sea region. Four different haplotypes were shared among the Finnhorse and Estonian local breeds (Fig. S2), with the predominating maternal line (haplogroup D3) pointing to global sharing and to genetic links between the Baltic Sea region and Siberian horses, specifically.

In conclusion, the studied breed set showed high variability in maternal inheritance, and mixed patterns of the international and native breeds of the Siberian and Baltic regions were revealed in the haplotype network. Further analysis is needed to determine the male‐specific phylogeny of Baltic horse breeds accurately.

All breeds in the Baltic Sea region (except the Estonian Heavy Draught breed) showed more maternal line variation compared with Siberian Altai and Yakutian horses, indicating multiple origins in the Baltic Sea region. Although the maternal variation of the ancestral population of Estonian breeds that served as a primary genetic resource during breed formation has been lost from the Estonian Native breed, it has been maintained by rare haplotype distribution within modern Tori horses. Matrilineal information could be used for better management of endangered breeds and for the amendment of conservation programmes.

## Supporting information


**Figure S1** Median joining network of haplogroups (nomenclature according to Cieslak *et al*. (2010), based on 43 haplotypes (calculated from 41 polymorphic sites, with four hotspots removed).
**Figure S2** Euler diagrams of Estonian and Finnish local breeds presenting (a) the number of horses according to their haplotype shared by or unique to different breeds and (b) the number of haplotypes common or unique to different breeds (EstN, Estonian Native Horse; EstH, Estonian Heavy Draught; Tori, Tori Horse; Finn, Finnhorse).
**Table S1** Sample information of studied horse breeds.
**Table S2** Primers used for Y‐chromosomal markers and mtDNA D‐loop sequence [between nucleotides 15 343 and 15 852 (509 bp; HVR1 region)].
**Table S3** MtDNA D‐loop HVR1 region variation and derived haplotypes (*N* = 72) based on 45 polymorphic sites.
**Table S4** Y‐chromosome microsatellite genotyping data.
**Table S5** Haplotypic distributions of mtDNA.
**Table S6** Occurrence of haplogroups (nomenclature used by Cieslak *et al*. 2010).
