# Supplementary material for: Maternal and paternal genetic variation in Estonian local horse breeds in the context of geographically adjacent and distant Eurasian breeds
Source: Anim Genet. 2019 Sep 2;50(6):757–60. doi: 10.1111/age.12835 (PMC6899971; doi:10.1111/age.12835)
Supplement: Supplementary file 1 — Figure S1 Median joining network of haplogroups (nomenclature according to Cieslak et al. (2010), based on 43 haplotypes (calculated from 41 polymorphic sites, with four hotspots removed). Figure S2 Euler diagrams of Estonian and Finnish local breeds presenting (a) the number of horses according to their haplotype shared by or unique to different breeds and (b) the number of haplotypes common or unique to different breeds (EstN, Estonian Native Horse; EstH, Estonian Heavy Draught; Tori, Tori Horse; Finn, Finnhorse). Table S1 Sample information of studied horse breeds. Table S2 Primers used for Y‐chromosomal markers and mtDNA D‐loop sequence [between nucleotides 15 343 and 15 852 (509 bp; HVR1 region)]. Table S3 MtDNA D‐loop HVR1 region variation and derived haplotypes (N = 72) based on 45 polymorphic sites. Table S4 Y‐chromosome microsatellite genotyping data. Table S5 Haplotypic distributions of mtDNA. Table S6 Occurrence of haplogroups (nomenclature used by Cieslak et al. 2010). [file AGE-50-757-s001.docx]

**Supporting Information**


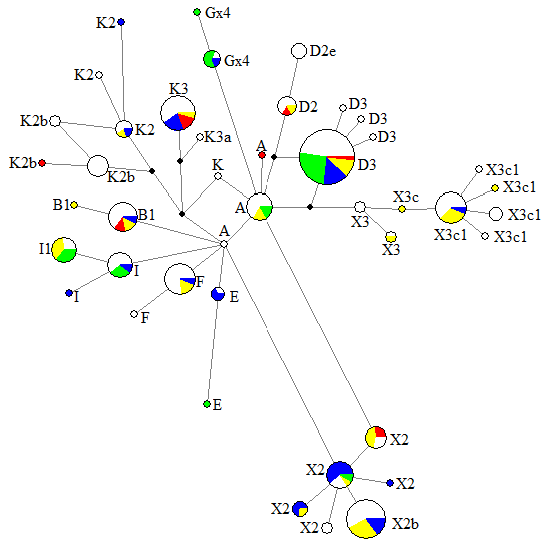


**Figure S1** Median joining network of haplogroups (nomenclature according to Cieslak et al. 2010), based on 43 haplotypes (calculated from 41 polymorphic sites, with four hotspots removed). The node area size corresponds to the number of individuals in the node. Red – Altai, yellow – Estonian Native, blue – Finnhorse, green – Yakutian, white – others (Trakehner, Arabian, Latvian).


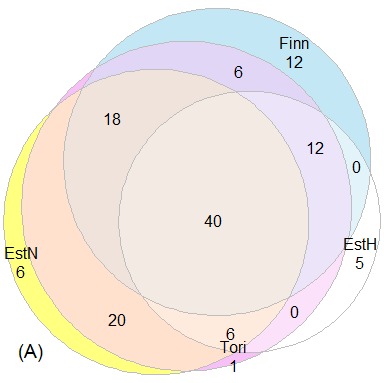

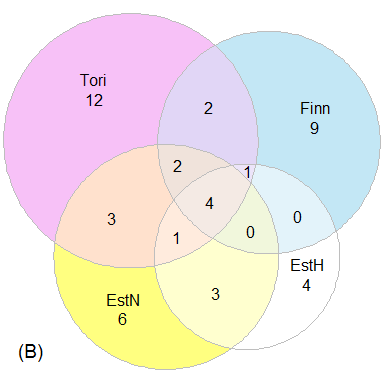


**Figure S2.** Euler diagrams of Estonian and Finnish local breeds presenting (A) the number of horses according to their haplotype shared by or unique to different breeds, (B) the number of haplotypes common or unique to different breeds (EstN – Estonian Native Horse, EstH – Estonian Heavy Draught, Tori – Tori Horse, Finn - Finnhorse).

**Table S1** Sample information of studied horse breeds.

| Breed | Abbreviation | N | ♂ | Breed type* | Sampling origin | GenBank number |
| --- | --- | --- | --- | --- | --- | --- |
| Altai | Alta | 11 | 9 | Native | Altai (Russian Federation) | MH794918 - MH794928 |
| Arabian | Arab | 29 | 19 | Non-native | Estonia | MH794889 – MH794917 |
| Estonian Heavy Draught | EstH | 30 | 12 | Non-native (EstN-derived) | Estonia | MH794769 – MH794798 |
| Estonian Native Horse | EstN | 40 | 31 | Native | Estonia | MH794849 – MH794888 |
| Finnhorse | Finn | 37 | 17 | Native | Finland | MH794731 – MH794767 |
| Latvian | Latv | 22 | 7 | Non-native | Latvia | MH794799 – MH794820 |
| Tori | Tori | 40 | 27 | Non-native (EstN-derived) | Estonia | MH794691 – MH794730 |
| Trakehner | Trak | 23 | 17 | Non-native | Estonia | MH794668 – MH794690 |
| Yakutian | Yaku | 27 | 13 | Native | Yakutia (Russian Federation) | MH794821 – MH794847 |

N – total number of sampled individuals

♂ - number of male samples

* “Native” indicates a subset of original locally adapted breeds without known recent introgression

**Table S2** Primers used for Y-chromosomal markers and mtDNA D-loop sequence (between nucleotides 15343 - 15852 (509 bp; HVR1 region)).

| Marker | Praimers | Reference |
| --- | --- | --- |
| YM2F | TGGTTCAGATGGTGTATTTTGTT | Wallner, *et al.,* 2004 |
| YM2R | TTTGCAGCCAGTACCTACCTT | Wallner, *et al.* 2004 |
| YH12F | CGAACAGGTGACGAAGCATC | Wallner, *et al.* 2004 |
| YH12R | GCAGACATGCACACCAACC | Wallner, *et al.* 2004 |
| YE1F | CTTCACTCCCGACCAAGAGA | Wallner, *et al.* 2004 |
| YE1R | GTGTGTCGTGCCGTGTTTAC | Wallner, *et al.* 2004 |
| YJ10F | AGTTCCCCTGCACACCT | Wallner, *et al.* 2004 |
| YJ10R | TGCCTCCCACAGCCATAC | Wallner, *et al.* 2004 |
| YA16F | TGACTGGAAATTGAAGATG | Wallner, *et al.* 2004 |
| YA16R | TTGTAGCAACAAAGTAACAC | Wallner, *et al.* 2004 |
| mtDNAF | CGCACATTACCCTGGTCTTG | Current study |
| mtDNAR | GAACCAGATGCCAGGTATAG | Current study |

**Table S3** MtDNA D-loop HVR1 region variation and derived haplotypes (N=72) based on 45 polymorphic sites.

| Haplotype | Haplogroup** | Nucleotide position | | | | | | | | | | | | | | | | | | | | | | | | | | | |  |  |  |  |  |  |  |  |  |  |  |  |  |  |  |  |  |
| --- | --- | --- | --- | --- | --- | --- | --- | --- | --- | --- | --- | --- | --- | --- | --- | --- | --- | --- | --- | --- | --- | --- | --- | --- | --- | --- | --- | --- | --- | --- | --- | --- | --- | --- | --- | --- | --- | --- | --- | --- | --- | --- | --- | --- | --- | --- |
|  |  | 4 | 4 | 4 | 4 | 4 | 5 | 5 | 5 | 5 | 5 | 5 | 5 | 5 | 5 | 5 | 5 | 6 | 6 | 6 | 6 | 6 | 6 | 6 | 6 | 6 | 6 | 6 | 6 | 6 | 6 | 6 | 6 | 7 | 7 | 7 | 7 | 7 | 7 | 7 | 7 | 7 | 7 | 7 | 7 | 7 |
|  |  | 8 | 9 | 9 | 9 | 9 | 2 | 2 | 2 | 3 | 3 | 3 | 4 | 5 | 8 | 9 | 9 | 0 | 0 | 0 | 0 | 0 | 1 | 3 | 3 | 4 | 5 | 5 | 5 | 5 | 6 | 6 | 7 | 0 | 0 | 2 | 2 | 3 | 4 | 4 | 7 | 7 | 7 | 7 | 8 | 8 |
|  |  | 5 | 0 | 4 | 5 | 6 | 0 | 1 | 8 | 2 | 4 | 8 | 2 | 2 | 5 | 6 | 7 | 0 | 1 | 2 | 3 | 4 | 7 | 2 | 5 | 9 | 0 | 2 | 7 | 9 | 6 | 7 | 6 | 3 | 9 | 0 | 6 | 7 | 0 | 7 | 0 | 1 | 5 | 7 | 2 | 7 |
| X79547* | D2e | G | C | C | C | G | A | G | C | C | T | A | C | T | A | A | A | G | T | T | C | A | T | T | C | G | A | T | T | T | G | A | A | T | C | A | G | T | A | A | C | C | C | A | T | A |
| H1 | K | A | . | . | C | . | . | . | . | . | . | . | . | . | . | . | . | A | . | T | . | A | . | . | . | . | . | . | . | . | . | . | . | C | . | A | . | . | G | . | . | T | . | G | . | . |
| H2 | X2b | . | . | C | C | G | . | . | . | . | T | . | . | . | A | . | . | . | . | . | C | . | . | . | . | G | . | . | . | . | . | . | . | . | . | A | . | . | . | . | . | T | . | . | . | . |
| H3 | X2 | . | . | C | C | G | . | . | . | . | T | . | . | . | A | . | . | . | . | T | C | A | . | . | . | G | . | . | . | . | . | . | . | . | . | A | . | . | . | . | . | . | . | . | . | . |
| H4 | X2 | . | . | C | C | G | . | . | . | . | T | . | . | . | A | . | . | . | . | T | C | A | . | . | . | G | . | . | . | . | . | . | . | . | . | A | . | . | . | . | . | T | . | . | . | . |
| H5 | X2b | . | . | C | C | G | . | . | . | . | T | . | . | . | . | . | . | . | . | . | C | . | . | . | . | G | . | . | . | . | . | . | . | . | . | A | . | . | . | . | . | T | . | . | . | . |
| H6 | X2 | . | . | C | C | G | . | . | . | . | T | . | . | . | . | . | . | . | . | T | C | . | . | . | . | G | . | . | . | . | . | . | . | . | . | A | . | . | . | . | . | T | . | . | . | . |
| H7 | X2 | . | . | C | C | G | . | . | . | . | T | . | . | . | . | . | . | . | . | T | C | . | . | . | . | G | . | . | . | . | . | . | . | . | . | A | . | . | . | . | . | T | . | . | . | G |
| H8 | X2 | . | . | C | C | G | . | . | . | . | T | . | . | . | . | . | . | . | . | T | C | . | . | . | . | G | . | . | . | . | . | . | . | . | . | A | . | . | . | . | T | T | . | . | . | . |
| H9 | X2 | . | . | C | C | G | . | . | T | . | T | . | . | . | A | . | . | . | . | T | C | . | . | . | . | G | . | . | . | . | . | . | . | . | . | A | . | . | . | . | . | T | . | . | . | . |
| H10 | X2 | . | . | C | C | G | . | . | T | . | T | . | . | . | . | . | . | . | . | T | C | . | . | . | . | G | . | . | . | . | . | . | . | . | . | A | . | . | . | . | . | T | . | . | . | . |
| H11 | Gx4 | . | . | . | C | . | . | A | . | . | . | . | . | . | A | . | . | . | . | T | . | . | . | . | . | . | G | . | . | . | . | . | . | . | . | A | . | C | . | . | T | . | . | . | . | . |
| H12 | D3 | . | . | . | C | . | . | C | . | . | . | . | . | . | A | . | G | . | . | . | . | . | . | . | . | . | G | . | . | . | A | . | . | . | . | A | . | . | . | . | . | . | . | . | . | . |
| H13 | Gx4 | . | . | . | C | . | . | A | . | . | . | . | . | . | A | . | G | . | . | T | . | . | . | . | . | . | . | . | . | . | . | . | . | . | . | A | . | C | . | . | T | . | . | . | . | . |
| H14 | Gx4 | . | . | . | C | . | . | A | . | . | . | . | . | . | A | . | G | . | . | T | . | . | . | . | . | . | G | . | . | . | . | . | . | . | . | A | . | C | . | . | T | . | . | . | . | . |
| H15 | Gx4 | . | . | . | C | . | . | A | . | . | . | . | . | . | . | . | . | . | . | T | . | . | . | . | . | . | . | . | . | . | . | . | . | . | . | A | . | C | . | . | T | . | . | . | . | . |
| H16 | X3c1 | . | . | . | C | . | . | A | . | . | . | . | T | . | . | . | G | . | . | T | . | . | . | . | T | . | G | . | . | . | A | . | . | C | . | A | . | . | . | . | . | . | . | . | . | . |
| H17 | K2 | . | . | . | C | . | . | . | . | . | . | . | . | C | A | . | . | . | . | T | . | A | . | . | . | . | . | . | . | . | . | . | . | C | . | A | . | . | G | . | . | T | . | G | . | . |
| H18 | F | . | . | . | C | . | . | . | . | . | . | . | . | . | A | . | . | . | C | T | . | . | . | . | . | . | . | . | . | . | . | . | . | . | . | A | . | . | . | . | . | T | . | . | . | . |
| H19 | D3 | . | . | . | C | . | . | . | . | . | . | . | . | . | A | . | . | . | . | . | . | . | . | . | . | . | G | . | . | . | A | . | . | . | . | A | . | . | . | . | . | . | . | . | . | . |
| H20 | K2 | . | . | . | C | . | . | . | . | . | . | . | . | . | A | . | . | . | . | T | . | A | . | . | . | . | . | . | . | . | . | . | . | C | . | A | . | . | G | . | . | T | . | G | . | . |
| H21 | K3 | . | . | . | C | . | . | . | . | . | . | . | . | . | A | . | . | . | . | T | . | A | . | . | . | . | . | . | . | . | . | G | . | C | . | A | . | . | . | . | . | T | . | G | . | . |
| H22 | B1 | . | . | . | C | . | . | . | . | . | . | . | . | . | A | . | . | . | . | T | . | . | C | . | . | . | . | . | . | C | . | . | . | . | . | A | . | . | . | . | . | T | . | . | . | . |
| H23 | A | . | . | . | C | . | . | . | . | . | . | . | . | . | A | . | . | . | . | T | . | . | . | . | . | . | . | . | . | . | . | . | . | . | . | A | . | . | . | . | . | T | . | . | . | . |
| H24 | K2 | . | . | . | C | . | . | . | . | . | . | . | . | . | A | . | G | . | . | T | . | A | . | . | . | . | . | . | . | . | . | . | . | C | . | A | . | . | G | . | . | T | . | G | . | . |
| H25 | K3 | . | . | . | C | . | . | . | . | . | . | . | . | . | A | . | G | . | . | T | . | A | . | . | . | . | . | . | . | . | . | G | . | C | . | A | . | . | . | . | . | T | . | G | . | . |
| H26 | F | . | . | . | C | . | . | . | . | . | . | . | . | . | . | . | . | . | C | T | . | . | . | . | . | . | . | . | . | . | . | . | . | . | . | A | . | . | . | . | . | T | . | . | C | . |
| H27 | F | . | . | . | C | . | . | . | . | . | . | . | . | . | . | . | . | . | C | T | . | . | . | . | . | . | G | . | . | . | . | . | . | . | . | A | . | . | . | . | . | T | . | . | . | . |
| H28 | D3 | . | . | . | C | . | . | . | . | . | . | . | . | . | . | . | . | . | . | . | . | . | C | . | . | . | G | . | . | . | A | . | . | . | . | A | . | . | . | . | . | . | . | . | . | . |
| H29 | D2 | . | . | . | C | . | . | . | . | . | . | . | . | . | . | . | . | . | . | . | . | . | . | . | . | . | . | . | . | . | . | . | . | . | . | . | . | . | . | . | . | . | . | . | . | . |
| H30 | D3 | . | . | . | C | . | . | . | . | . | . | . | . | . | . | . | . | . | . | . | . | . | . | . | . | . | G | . | . | . | A | . | . | . | . | A | . | . | . | . | . | . | . | . | . | . |
| H31 | K2b | . | . | . | C | . | . | . | . | . | . | . | . | . | . | . | . | . | . | T | . | A | . | . | . | . | . | . | . | . | . | . | . | C | . | A | A | . | G | . | . | T | . | . | . | . |
| H32 | K2 | . | . | . | C | . | . | . | . | . | . | . | . | . | . | . | . | . | . | T | . | A | . | . | . | . | . | . | . | . | . | . | . | C | . | A | . | . | G | . | . | T | . | G | . | . |
| H33 | K3 | . | . | . | C | . | . | . | . | . | . | . | . | . | . | . | . | . | . | T | . | A | . | . | . | . | . | . | . | . | . | G | . | C | . | A | . | . | . | . | . | T | . | G | . | . |
| H34 | K2 | . | . | . | C | . | . | . | . | . | . | . | . | . | . | . | . | . | . | T | . | A | . | . | . | . | G | . | . | . | . | . | . | C | . | A | . | . | G | . | . | T | . | G | . | . |
| H35 | A | . | . | . | C | . | . | . | . | . | . | . | . | . | . | . | . | . | . | T | . | A | . | . | . | . | G | . | . | . | . | . | . | . | . | A | . | . | . | . | . | . | . | . | . | . |
| H36 | B1 | . | . | . | C | . | . | . | . | . | . | . | . | . | . | . | . | . | . | T | . | . | C | . | . | . | . | . | . | C | . | . | . | . | . | A | . | . | . | . | . | T | . | . | C | . |
| H37 | B1 | . | . | . | C | . | . | . | . | . | . | . | . | . | . | . | . | . | . | T | . | . | C | . | . | . | . | . | . | C | . | . | . | . | . | A | . | . | . | . | . | T | . | . | . | . |
| H38 | K2b | . | . | . | C | . | . | . | . | . | . | . | . | . | . | . | . | . | . | T | . | . | . | . | . | . | . | . | . | . | . | . | . | C | . | A | A | . | G | . | . | T | . | G | . | . |
| H39 | A | . | . | . | C | . | . | . | . | . | . | . | . | . | . | . | . | . | . | T | . | . | . | . | . | . | . | . | . | . | . | . | . | . | . | A | . | . | . | . | . | . | . | . | . | . |
| H40 | A | . | . | . | C | . | . | . | . | . | . | . | . | . | . | . | . | . | . | T | . | . | . | . | . | . | G | C | . | . | . | . | . | . | . | A | . | . | . | . | . | . | . | . | . | . |
| H41 | K | . | . | . | C | . | . | . | . | . | . | . | . | . | . | . | . | . | . | T | . | . | . | . | . | . | G | . | . | . | . | . | . | C | . | A | . | . | . | . | . | . | . | . | . | . |
| H42 | A | . | . | . | C | . | . | . | . | . | . | . | . | . | . | . | . | . | . | T | . | . | . | . | . | . | G | . | . | . | . | . | . | . | . | A | . | . | . | . | . | . | . | . | . | . |
| H43 | D3 | . | . | . | C | . | . | . | . | . | . | . | . | . | . | . | G | . | . | . | . | . | . | . | . | . | G | . | . | . | A | . | . | . | . | A | . | . | . | . | . | . | . | . | . | . |
| H44 | K2b | . | . | . | C | . | . | . | . | . | . | . | . | . | . | . | G | . | . | T | . | A | . | . | . | . | . | . | . | . | . | . | . | C | . | A | A | . | G | . | . | T | . | . | . | . |
| H45 | K2b | . | . | . | C | . | . | . | . | . | . | . | . | . | . | . | G | . | . | T | . | A | . | . | . | . | . | . | . | . | . | . | . | C | . | A | A | . | G | G | . | T | . | . | . | . |
| H46 | K3 | . | . | . | C | . | . | . | . | . | . | . | . | . | . | . | G | . | . | T | . | A | . | . | . | . | . | . | . | . | . | G | . | C | . | A | . | . | . | . | . | T | . | G | . | . |
| H47 | K3a | . | . | . | C | . | . | . | . | . | . | . | . | . | . | . | G | . | . | T | . | A | . | . | T | . | . | . | . | . | . | G | . | C | . | A | . | . | . | . | . | T | . | . | . | . |
| H48 | B1 | . | . | . | C | . | . | . | . | . | . | . | . | . | . | . | G | . | . | T | . | . | C | . | . | . | . | . | . | C | . | . | . | . | . | A | . | . | . | . | . | T | . | . | . | . |
| H49 | A | . | . | . | C | . | . | . | . | . | . | . | . | . | . | . | G | . | . | T | . | . | . | . | . | . | . | . | . | . | . | . | . | . | . | A | . | . | . | . | . | . | . | . | . | . |
| H50 | A | . | . | . | C | . | . | . | . | . | . | . | . | . | . | . | G | . | . | T | . | . | . | . | . | . | G | . | . | . | . | . | . | . | . | A | . | . | . | . | . | . | . | . | . | . |
| H51 | X3 | . | . | . | C | . | . | . | . | . | . | . | T | . | A | . | . | . | . | T | . | . | . | . | . | . | G | . | . | . | A | . | . | . | . | A | . | . | . | . | . | . | . | . | . | . |
| H52 | X3 | . | . | . | C | . | . | . | . | . | . | . | T | . | A | . | . | . | . | T | . | . | . | . | . | . | G | . | . | . | A | . | . | . | . | A | . | . | . | . | . | T | . | . | . | . |
| H53 | X3 | . | . | . | C | . | . | . | . | . | . | . | T | . | A | . | G | . | . | T | . | . | . | . | . | . | G | . | . | . | A | . | . | . | . | A | . | . | . | . | . | T | . | . | . | . |
| H54 | X3c1 | . | . | . | C | . | . | . | . | . | . | . | T | . | A | . | G | . | . | T | . | . | . | . | T | . | G | . | . | . | A | . | . | C | . | A | . | . | . | . | . | . | . | . | . | . |
| H55 | X3c1 | . | . | . | C | . | . | . | . | . | . | . | T | . | A | . | G | . | . | T | . | . | . | . | T | . | G | . | . | . | A | . | . | C | . | A | . | . | . | . | . | . | T | . | . | . |
| H56 | X3c1 | . | . | . | C | . | . | . | . | . | . | . | T | . | . | . | G | . | . | T | . | . | . | C | T | . | . | . | . | . | A | . | . | C | . | A | . | . | . | . | . | . | . | . | . | . |
| H57 | X3c1 | . | . | . | C | . | . | . | . | . | . | . | T | . | . | . | G | . | . | T | . | . | . | . | T | . | G | . | . | . | A | . | . | C | . | A | . | . | . | . | . | . | . | . | . | . |
| H58 | X3c1 | . | . | . | C | . | . | . | . | . | . | . | T | . | . | . | G | . | . | T | . | . | . | . | T | . | G | . | . | . | A | . | . | C | . | A | . | . | . | . | . | . | T | . | . | . |
| H59 | X3c1 | . | . | . | C | . | . | . | . | . | . | . | T | . | . | . | G | . | . | T | . | . | . | . | T | . | G | . | . | . | A | . | . | C | . | A | . | . | . | . | T | . | . | . | . | . |
| H60 | X3c | . | . | . | C | . | . | . | . | . | . | . | T | . | . | . | G | . | . | T | . | . | . | . | T | . | G | . | . | . | A | . | . | . | . | A | . | . | . | . | . | . | . | . | . | . |
| H61 | I | . | . | . | C | . | . | . | . | . | . | G | . | . | A | . | . | A | . | T | . | A | . | . | . | . | G | . | . | . | . | . | . | . | T | A | . | . | . | . | . | T | . | . | . | . |
| H62 | I | . | . | . | C | . | . | . | . | . | . | G | . | . | A | . | . | . | . | T | . | . | . | . | . | . | . | . | . | . | . | . | . | . | T | A | . | . | . | . | . | T | . | . | . | . |
| H63 | I | . | . | . | C | . | . | . | . | . | . | G | . | . | A | . | . | . | . | T | . | . | . | . | . | . | G | . | . | . | . | . | . | . | T | A | . | . | . | . | . | T | . | . | . | . |
| H64 | I | . | . | . | C | . | . | . | . | . | . | G | . | . | A | . | G | . | . | T | . | . | . | . | . | . | G | . | . | . | . | . | . | . | T | A | . | . | . | . | . | T | . | . | . | . |
| H65 | I | . | . | . | C | . | . | . | . | . | . | G | . | . | . | . | G | . | . | T | . | . | . | . | . | . | G | . | . | . | . | . | . | . | T | A | . | . | . | . | . | T | . | . | . | . |
| H66 | I1 | . | . | . | C | . | . | . | . | . | . | G | . | . | . | G | . | . | . | T | . | . | . | . | . | . | . | . | . | . | . | . | . | . | T | A | . | . | . | . | . | T | . | . | . | . |
| H67 | I1 | . | . | . | C | . | . | . | . | . | . | G | . | . | . | G | . | . | . | T | . | . | . | . | . | . | G | . | . | . | . | . | . | . | T | A | . | . | . | . | . | T | . | . | . | . |
| H68 | E | . | . | . | C | . | . | . | . | - | . | . | . | . | A | . | . | . | . | T | . | . | . | . | . | . | . | . | C | . | . | . | G | . | . | A | . | . | . | . | . | T | . | . | . | . |
| H69 | E | . | . | . | C | . | . | . | . | - | . | . | . | . | A | . | . | . | . | T | . | . | . | . | . | . | . | . | . | . | . | . | . | . | . | A | . | . | . | . | . | T | . | . | . | . |
| H70 | Gx4 | . | . | . | C | . | G | A | . | . | . | . | . | . | A | . | . | . | . | T | . | . | . | . | . | . | . | . | . | . | . | . | . | . | . | A | . | C | . | . | T | . | . | . | . | . |
| H71 | D2e | . | . | . | . | . | . | . | . | . | . | . | . | . | . | . | . | . | . | . | . | . | . | . | . | . | . | . | . | . | . | . | . | . | . | . | . | . | . | . | . | . | . | . | . | . |
| H72 | D3 | . | T | . | C | . | . | . | . | . | . | . | . | . | . | . | . | . | . | . | . | . | . | . | . | . | G | . | . | . | A | . | . | . | . | A | . | . | . | . | . | . | . | . | . | . |

*- nucleotide positions are defined by reference sample presented by Xu and Arnason 1994

** - nomenclature by Cieslak *et al*. 2010

|  |
| --- |
|  |

**Table S4** Y-chromosome microsatellite genotyping data.

| Breed | N | YM2 | YH12 | YE1 | YA16 | YJ10 |
| --- | --- | --- | --- | --- | --- | --- |
| Altai | 9 | 116 | 100 | 196 | 157(A) | 213 |
| Arabian | 19 | 116 | 100 | 196 | 157(A) | 213 |
| Estonian Native | 31 | 116 | 100 | 196 | 157(A) | 213 |
| Yakutian | 9 | 116 | 100 | 196 | 157(A) | 213 |
| Yakutian | 4 | 116 | 100 | 196 | 153(B) | 213 |
| Latvian | 7 | 116 | 100 | 196 | 157(A) | 213 |
| Estonian Heavy Draught | 12 | 116 | 100 | 196 | 157(A) | 213 |
| Finnhorse | 17 | 116 | 100 | 196 | 157(A) | 213 |
| Tori | 27 | 116 | 100 | 196 | 157(A) | 213 |
| Trakehner | 17 | 116 | 100 | 196 | 157(A) | 213 |

**Table S5** Haplotypic distributions of mtDNA.

| Haplotype | EstH | Tori | EstN | Finn | Alta | Yaku | Arab | Latv | Trak | All |
| --- | --- | --- | --- | --- | --- | --- | --- | --- | --- | --- |
| H1 |  |  |  | 1 |  |  |  |  |  | 1 |
| H2 |  | 3 | 2 | 4 |  |  | 4 | 2 | 3 | 18 |
| H3 | 1 |  | 3 |  | 2 |  |  | 1 |  | 7 |
| H4 |  |  |  | 1 |  | 1 |  |  |  | 2 |
| H5 |  | 2 | 5 |  |  |  |  |  | 1 | 8 |
| H6 |  | 1 | 1 | 7 |  |  |  | 2 |  | 11 |
| H7 |  |  |  | 1 |  |  |  |  |  | 1 |
| H8 |  | 1 |  |  |  |  |  |  | 1 | 2 |
| H9 |  |  | 1 |  |  |  |  |  |  | 1 |
| H10 |  |  |  | 3 |  |  |  |  |  | 3 |
| H11 | 1 |  |  |  |  |  |  |  |  | 1 |
| H12 |  | 1 |  |  |  |  |  |  |  | 1 |
| H13 |  |  |  |  |  | 1 |  |  |  | 1 |
| H14 |  |  |  |  |  | 2 |  |  |  | 2 |
| H15 |  |  |  | 1 |  |  |  |  |  | 1 |
| H16 |  |  | 1 |  |  |  |  |  |  | 1 |
| H17 |  |  |  |  |  |  |  | 1 |  | 1 |
| H18 | 9 | 1 | 3 | 1 |  |  |  | 1 | 1 | 16 |
| H19 | 2 | 4 |  | 6 |  |  | 3 |  | 2 | 17 |
| H20 |  |  | 1 |  |  |  |  |  |  | 1 |
| H21 |  |  |  | 1 |  |  |  |  | 1 | 2 |
| H22 |  | 1 |  |  |  |  |  | 1 | 1 | 3 |
| H23 | 1 |  |  |  |  |  |  |  |  | 1 |
| H24 |  |  |  |  |  |  |  | 1 |  | 1 |
| H25 |  |  |  |  | 3 |  |  |  |  | 3 |
| H26 |  |  |  |  |  |  |  | 1 |  | 1 |
| H27 |  |  |  |  |  |  |  | 1 |  | 1 |
| H28 |  |  |  |  |  |  |  | 1 |  | 1 |
| H29 | 1 |  | 1 |  | 1 |  | 1 |  | 2 | 6 |
| H30 | 6 | 1 | 5 | 1 | 1 | 12 | 3 |  |  | 29 |
| H31 |  |  |  |  |  |  |  |  | 1 | 1 |
| H32 |  | 1 |  | 1 |  |  |  |  |  | 2 |
| H33 | 1 |  | 1 |  |  |  |  |  | 1 | 3 |
| H34 |  |  |  |  |  |  |  |  | 1 | 1 |
| H35 |  |  |  |  |  | 1 |  |  |  | 1 |
| H36 |  |  | 1 |  |  |  |  |  |  | 1 |
| H37 | 1 | 3 | 2 | 1 | 2 |  | 1 |  |  | 10 |
| H38 |  |  |  |  |  |  |  |  | 2 | 2 |
| H39 | 2 |  |  |  |  | 1 |  |  |  | 3 |
| H40 |  |  |  |  | 1 |  |  |  |  | 1 |
| H41 |  | 1 |  |  |  |  |  |  |  | 1 |
| H42 |  |  |  |  |  |  |  |  | 1 | 1 |
| H43 |  |  |  |  |  |  |  | 2 |  | 2 |
| H44 |  |  |  |  |  |  | 6 |  |  | 6 |
| H45 |  |  |  |  | 1 |  |  |  |  | 1 |
| H46 |  | 1 |  | 3 |  |  | 6 | 2 |  | 12 |
| H47 |  |  |  |  |  |  |  |  | 1 | 1 |
| H48 |  | 1 |  |  |  |  |  |  |  | 1 |
| H49 |  | 1 |  |  |  |  |  |  |  | 1 |
| H50 |  | 4 | 2 |  |  |  |  |  |  | 6 |
| H51 |  | 2 |  |  |  |  |  |  |  | 2 |
| H52 |  |  | 1 |  |  |  |  |  |  | 1 |
| H53 |  | 1 |  |  |  |  |  |  |  | 1 |
| H54 | 3 | 1 | 1 | 1 |  |  |  |  | 1 | 7 |
| H55 | 1 |  |  |  |  |  |  |  |  | 1 |
| H56 |  | 1 |  |  |  |  |  |  |  | 1 |
| H57 | 1 | 1 | 4 |  |  |  | 1 | 2 |  | 9 |
| H58 |  | 2 |  |  |  |  |  |  |  | 2 |
| H59 |  |  |  |  |  |  |  | 1 |  | 1 |
| H60 |  |  | 1 |  |  |  |  |  |  | 1 |
| H61 |  |  |  | 1 |  |  |  |  |  | 1 |
| H62 |  |  |  |  |  | 2 |  | 1 |  | 3 |
| H63 |  |  |  |  |  | 1 | 4 |  |  | 5 |
| H64 |  |  |  | 1 |  |  |  |  |  | 1 |
| H65 |  | 1 |  |  |  |  |  |  |  | 1 |
| H66 |  |  |  |  |  | 4 |  |  |  | 4 |
| H67 |  | 3 | 4 |  |  |  |  |  |  | 7 |
| H68 |  |  |  |  |  | 1 |  |  |  | 1 |
| H69 |  |  |  | 2 |  |  |  | 1 |  | 3 |
| H70 |  |  |  |  |  | 1 |  |  |  | 1 |
| H71 |  | 1 |  |  |  |  |  |  | 3 | 4 |
| H72 |  |  |  |  |  |  |  | 1 |  | 1 |
| Count | 13 | 25 | 19 | 18 | 7 | 11 | 9 | 17 | 16 | 72 |

**Table S6** Occurrence of haplogroups (nomenclature used by Cieslak *et al*., 2010).

| Haplogroup | Alta | Arab | EstN | Latv | EstH | Finn | Tori | Trak | Yaku | All |
| --- | --- | --- | --- | --- | --- | --- | --- | --- | --- | --- |
| A | 1 |  | 2 |  | 3 |  | 5 | 1 | 2 | 14 |
| B1 | 2 | 1 | 3 | 1 | 1 | 1 | 5 | 1 |  | 15 |
| D2 | 1 | 1 | 1 |  | 1 |  |  | 2 |  | 6 |
| D2e |  |  |  |  |  |  | 1 | 3 |  | 4 |
| D3 | 1 | 6 | 5 | 4 | 8 | 7 | 6 | 2 | 12 | 51 |
| E |  |  |  | 1 |  | 2 |  |  | 1 | 4 |
| F |  |  | 3 | 3 | 9 | 1 | 1 | 1 |  | 18 |
| Gx4 |  |  |  |  | 1 | 1 |  |  | 4 | 6 |
| I |  | 4 |  | 1 |  |  | 2 | 1 | 3 | 11 |
| I1 |  |  | 4 |  |  |  | 3 |  | 4 | 11 |
| K |  |  |  |  |  |  | 1 |  |  | 1 |
| K2 |  |  | 1 | 2 |  | 2 | 1 | 1 |  | 7 |
| K2b | 1 | 6 |  |  |  |  |  | 3 |  | 10 |
| K3 | 3 | 6 | 1 | 2 | 1 | 4 | 1 | 3 |  | 21 |
| K3a |  |  |  |  |  |  |  | 1 |  | 1 |
| X2 | 2 |  | 5 | 3 | 1 | 12 | 2 | 1 | 1 | 37 |
| X2b |  | 4 | 7 | 2 |  | 4 | 5 | 4 |  | 26 |
| X3 |  |  | 1 |  |  |  | 3 |  |  | 4 |
| X3c |  |  | 1 |  |  |  |  |  |  | 1 |
| X3c1 |  | 1 | 6 | 3 | 5 | 1 | 5 | 1 |  | 22 |
| Count | 7 | 8 | 13 | 10 | 9 | 10 | 14 | 14 | 7 |  |
